# Supplementary material for: Revisiting co-expression-based automated function prediction in yeast with neural networks and updated Gene Ontology annotations
Source: PLoS One. 2026 Apr 16;21(4):e0322689. doi: 10.1371/journal.pone.0322689 (PMC13086303; doi:10.1371/journal.pone.0322689)
Supplement: S1 Text — A document containing additional analysis and supplemental figures relating to our methodology and results. The supplemental methods contain sections about Pearson correlation calculation adjustments, model hyperparameters and training, an analysis of overfitting, and a time complexity analysis. The supplemental results contain an analysis of our methodology’s sensitivity to initialization parameters and the cross-validation data split, and it contains a figure analyzing the relationship between model performance and a GO term’s co-expression z-score distribution. (PDF) [file pone.0322689.s001.pdf]

S1. Supplementary methods

S1.1 Addressing corner cases in Pearson correlation calculations

For certain gene pairs, the Pearson correlation is uninformative of their co-expression relationship or prevents the use of a Fisher transformation. Therefore, an adjusted Pearson calculation $\rho'_{x,y}$ is calculated as:

$$\rho'_{x,y} = \begin{cases} 0 & \text{if } C(x,y) < \frac{n}{2}, \\ 0.99 & \text{if } \rho_{x,y} = 1, \\ -0.99 & \rho_{x,y} = -1, \\ \rho_{x,y} & \text{otherwise} \end{cases}$$

where $C(x_i, y_i)$ is the number of conditions such that both genes x and y both have recorded gene expression values. We found that when half of the experimental conditions lack gene expression values for either of the genes in a pair, the resulting Pearson correlation was uninformative of how similar the gene expression of the pair is in real biological systems. Therefore, these Pearson correlations are adjusted to 0, which is intended to represent that the gene pair is not known to have correlated gene expression. Additionally, Pearson correlation values of 1 and -1 are adjusted to 0.99 and -0.99 respectively because the Fisher Z transformation is undefined at values 1 and -1.

S1.2 Model hyperparameters, loss curves, and implementation hardware

We trained the following four models for various comparisons in our results:

Model Number	Description	Section referenced in	Input size	Hidden layer sizes	Output size
1	Trained for best performance across all GO terms, used in comparison to MEFIT and SPELL	3.1 and 3.2	113	500, 200, 100	79
2	Model trained with 2007 gene expression data and 2007 Gene Ontology annotations	3.3	113	80, 80, 80	79
3	Model trained with 2022 gene expression data and 2007 Gene Ontology annotations	3.3	430	80, 80, 80	93
4	Model trained with 2022 gene expression data and 2022 Gene Ontology annotations	3.3	430	80, 80, 80	93

Table A. Summary of Models Referenced in Results

All models trained in this paper used a binary cross entropy loss function and a ReLU activation function. All models were trained with the following hyperparameters found in Table 2S on a server with Intel Xeon CPU E5-2695 v4 2.10GHz, CentOS Linux 7, Python 3.9.12, and PyTorch 1.13.1.

learning rate	0.01
momentum	0.9
batch size	50

training length	600,000 batches

Table B. Hyperparameters used for the training of all neural networks listed in this paper

After training each model's 4 cross-validated neural networks for 600,000 training batches, we found that the training loss of each network had converged (S1 Fig). We note that overfitting—the difference between testing and training loss measured by the binary cross-entropy loss function—tends to increase the longer each network is trained. To determine how this overfitting impacts downstream performance, we trained eight models with different training-testing splits and evaluated their performance at multiple steps in training (S2 Fig). Despite overfitting increasing with the number of batches trained, the average pairwise testing AUC and single gene AUC across all GO terms only increase over training time. Therefore, while our models overfit on their binary cross-entropy training objective more over training time, this does not negatively impact our downstream performance.

This apparent discrepancy likely reflects a mismatch between the optimization objective and the evaluation metrics: cross-entropy loss penalizes confident misclassification of individual training examples, including mislabeled or noisy examples, whereas ROC and ranking-based evaluations emphasize relative ordering rather than probability calibration [1-3]. In the presence of label noise (such as the mis-annotated negatives in the Gene Ontology discussed in Section 3.2 of the main text), continued training may therefore improve ranking performance even as loss increases. This trend can be seen in Fig S2 where single gene AUC continues to improve even after pure testing metrics have leveled off or worsened.

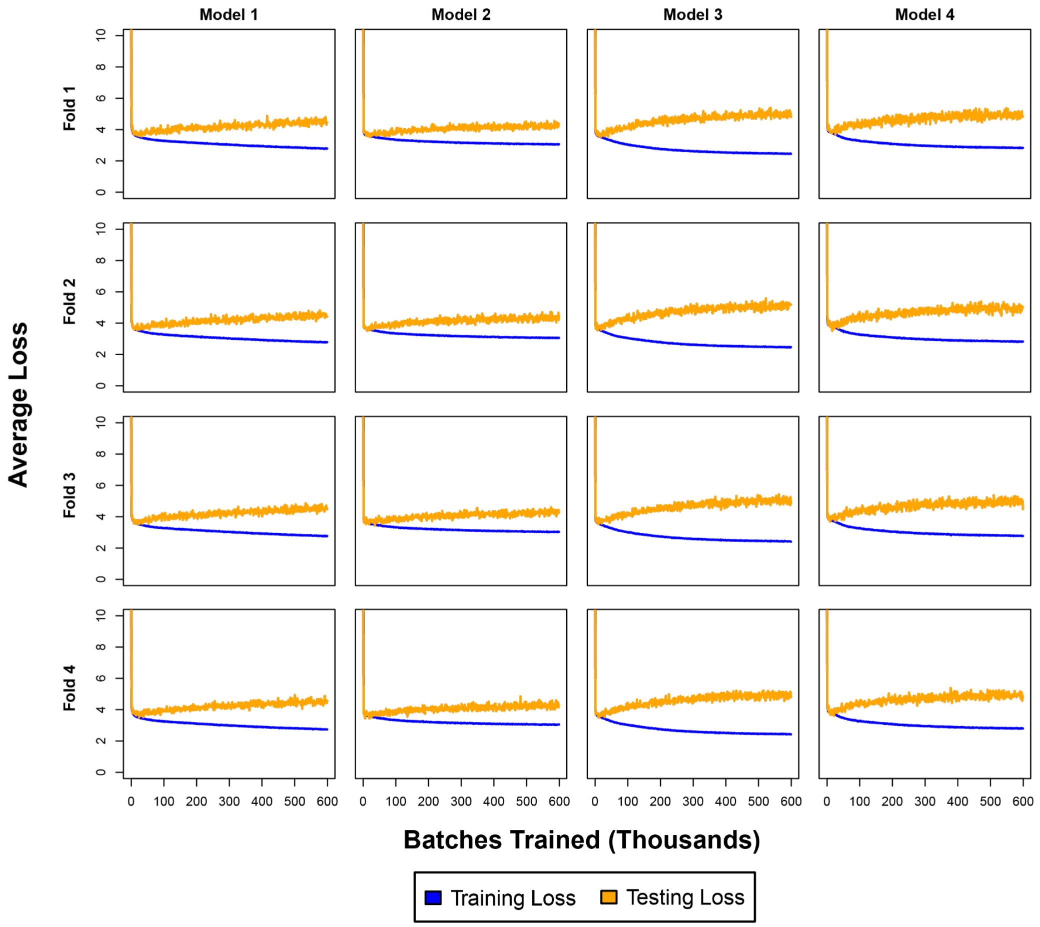

S1 Fig. Loss Curves of Cross-validated Models. Line plot of the number of batches trained on against the average binary cross entropy loss of every 1000 training data batches and the average loss of 100 testing data batches. Each column represents a different model listed in Table S2. Each row represents a different fold (a neural network trained on a different testing and training data split).

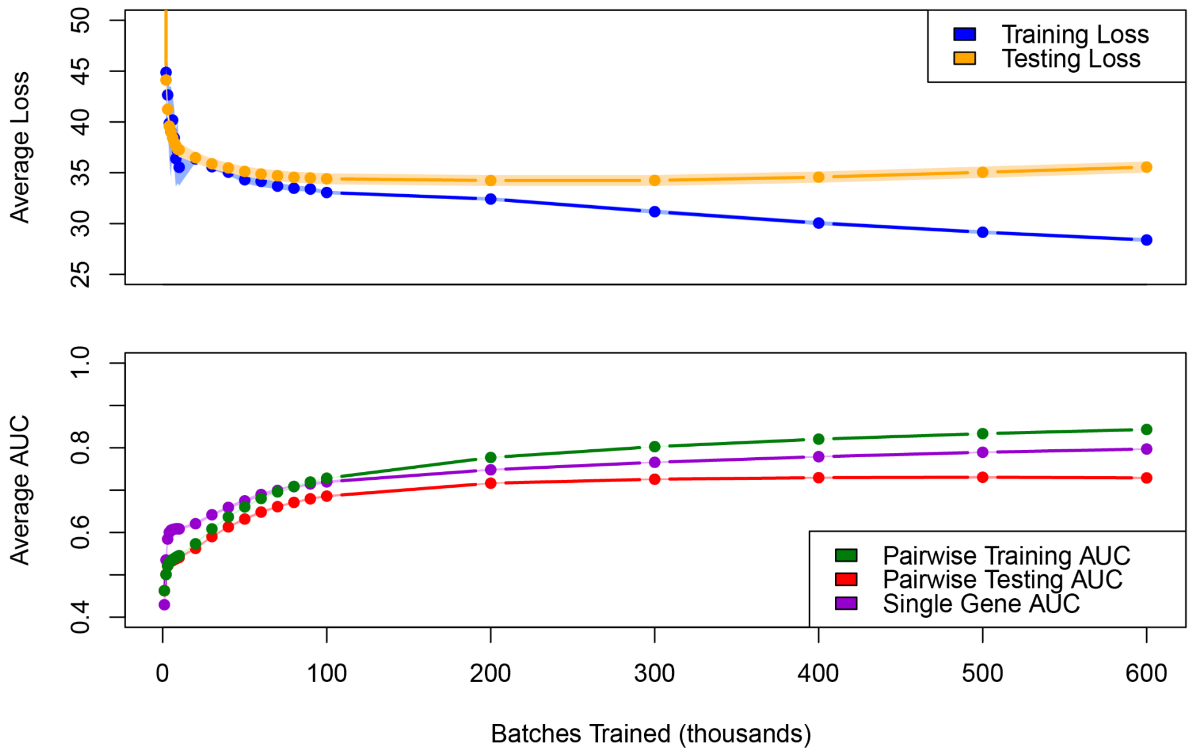

S2 Fig. Relationship Between Overfitting and Downstream Performance. Line plot displaying mean training loss, testing loss, and three AUC performance metrics over the course of training, averaged across 8 replicate models. The highlighted portion of each line represents the 95% confidence interval; however, it is too small to see for most lines. All AUC metrics are averaged across all 79 predicted GO terms, and pairwise AUC metrics derived by averaging across all four folds of each model

S1.3 Time complexity analysis

In terms of training a neural network, the feedforward and backpropagation algorithms have a linear time complexity with respect to the number of trainable weights [4]. We find our model is most performant with three hidden layers where the widest hidden layer has within an

order of magnitude of input size number of hidden neurons. Therefore, the number of trainable weights scales quadratically with respect to the number of input features *n*. To train our network, we perform *i* iterations of the training loop where each interaction performs a forward and backwards pass on *b* samples in a mini-batch. Therefore, the complexity of training our model is $O(n^2bi)$.

To generate a ranked prediction of *g* genes involved in a particular GO term, we first perform a forward pass ($O(n^2)$ operations) for each pair of genes ($O(g^2)$ genes pairs). Therefore, this process requires $O(n^2g^2)$ operations. Next, we calculate the sum of each gene's connections to all genes annotated to the given GO term. As a GO term's annotated genes are a subset of all the genes we are ranking, this requires $O(g^2)$ operations. Altogether, the time complexity of our evaluation algorithm is $O(n^2g^2)$.

S2. Supplementary results

S2.1 Sensitivity of model to starting parameters and subset randomization

To determine the sensitivity of our model's performance to the neural networks' starting parameters and the random division of our gold standard genes into subsets, we trained 20 models with the same random gold standard subsets and different neural network parameter initializations, and we trained 20 models with different random gold standard subsets. We evaluated each model's performance on pairwise rankings, pairwise rankings with each model's folds averaged, and single gene rankings (S3 Fig). Percent change over random precision (PCORP) is calculated as the percent change in each ranking's average precision compared to

random precision for that ranking. Note that for GO terms with few annotations, some randomly initialized folds lacked enough positive annotations to generate positive gene pairs, so there are no evaluations for those folds.

We found that the patterns of variability for each of our kinds of performance evaluations across each GO term was similar between models trained with different starting parameters and models trained with different gold standard subsets. We found that AUC's rankings of gene pairs predicted to be involved in each GO term were highly variable across folds (S3.A Fig), but when averaging the folds of the same model together, this variability is greatly reduced (S3.B Fig). This suggests that folds that perform worse than average for a given GO term are compensated by one or more folds from the same model that perform better than average. Furthermore, the AUC's from rankings of individual genes for each GO term also had a low variability (S3.C Fig). This suggests that the final rankings of genes involved in each term are robust to changes in starting parameters and the randomization of gold standard subsets.

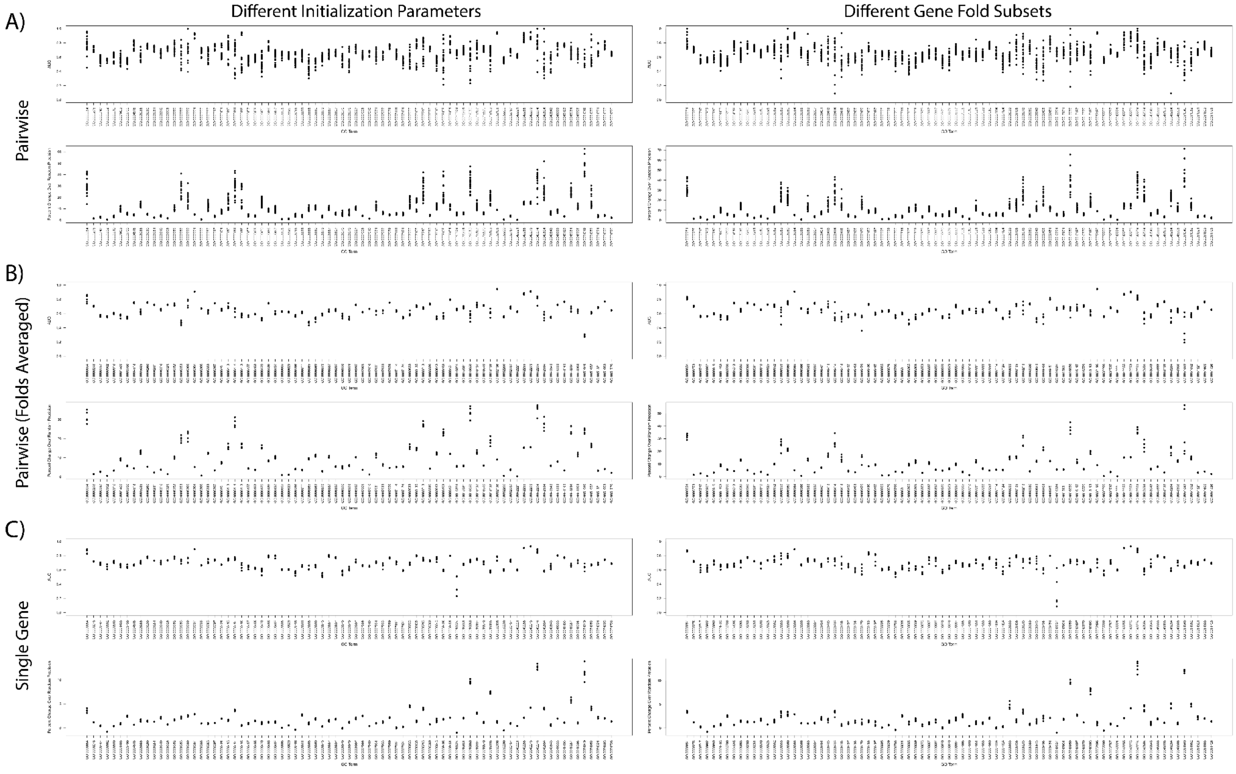

S3 Fig. Consistency of model performance with different initialization parameters and random gene folds. Strip plots plotting the AUCs and percent change over random precisions (PCORP) for each GO term. The left column shows the distribution of AUCs and PCORP when training models with different neural network starting parameters. The right column shows the distribution of AUCs and PCORP when training models with different random gold standard subsets. Performance is evaluated for three different kinds of predictions (A) Pairwise rankings of testing gene pairs for each fold of each model. (B) Same pairwise rankings with the folds of each model averaged together for each GO term. (C) Single gene ranking from each GO term's functional relationship graphs.

S2.2 Relationship between GO term performance and co-expression z-score distribution

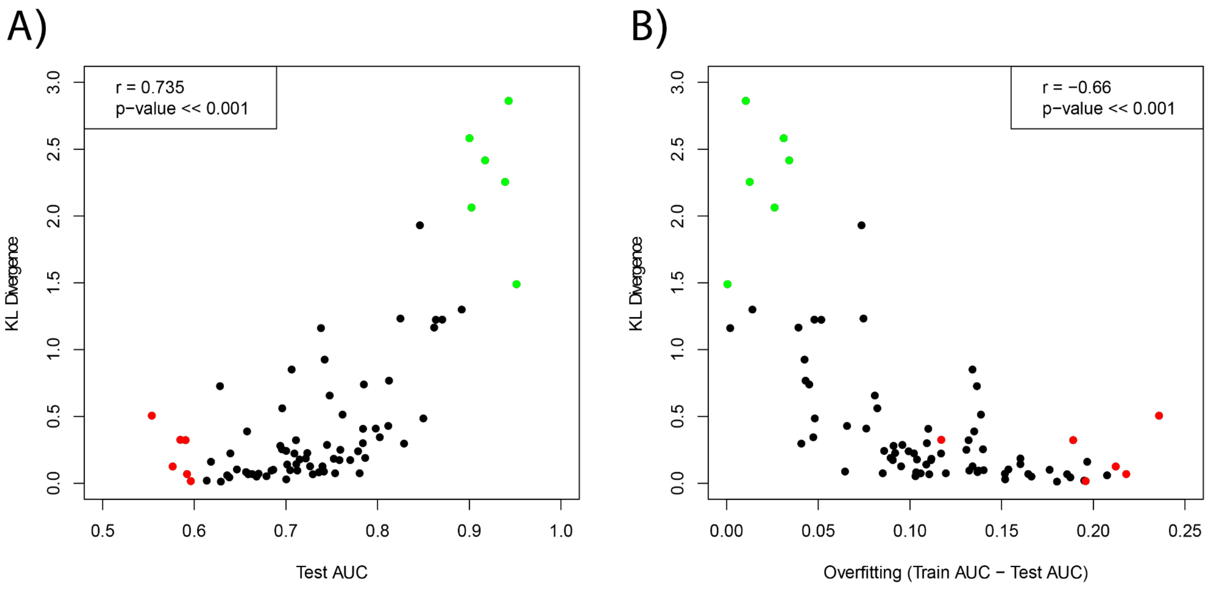

S4 Fig. Relationship between GO term performance and difference of pairwise co-expression distribution from background. For each GO term, we derived a probability of the average z-score across 113 gene expression datasets for a sample of 200,000 of the term's co-

annotated pairs. We measured the KL divergence between this distribution and the background probability distribution from the average z-scores of 200,000 gene pairs sampled from the set of all training gene pairs. The GO terms denoted by green and red are the same GO terms that are denoted by the green and red circles in Fig 3. (A) KL divergence plotted against test AUC for each GO term ($r = 0.848$; $p << 0.001$). (B) KL divergence plotted against overfitting (defined as training AUC - test AUC) for each GO term (-0.622; $p << 0.001$). This figure was constructed using data found in S1 Table.

Supplemental References

1. Yuan Z, Yan Y, Sonka M, Yang T. Large-scale robust deep AUC maximization: A new surrogate loss and empirical studies on medical image classification. arXiv [cs.LG]. 2020. doi:10.48550/arXiv.2012.03173

2. Wang Y, Ma X, Chen Z, Luo Y, Yi J, Bailey J. Symmetric Cross Entropy for robust learning with noisy labels. arXiv [cs.LG]. 2019. doi:10.48550/arXiv.1908.06112

3. Byrne S. Empirical AUC for evaluating probabilistic forecasts. arXiv [math.ST]. 2015. doi:10.48550/arXiv.1508.05503

4. Aggarwal CC. Neural networks and deep learning: A textbook. Cham: Springer International Publishing; 2018. doi:10.1007/978-3-319-94463-0
